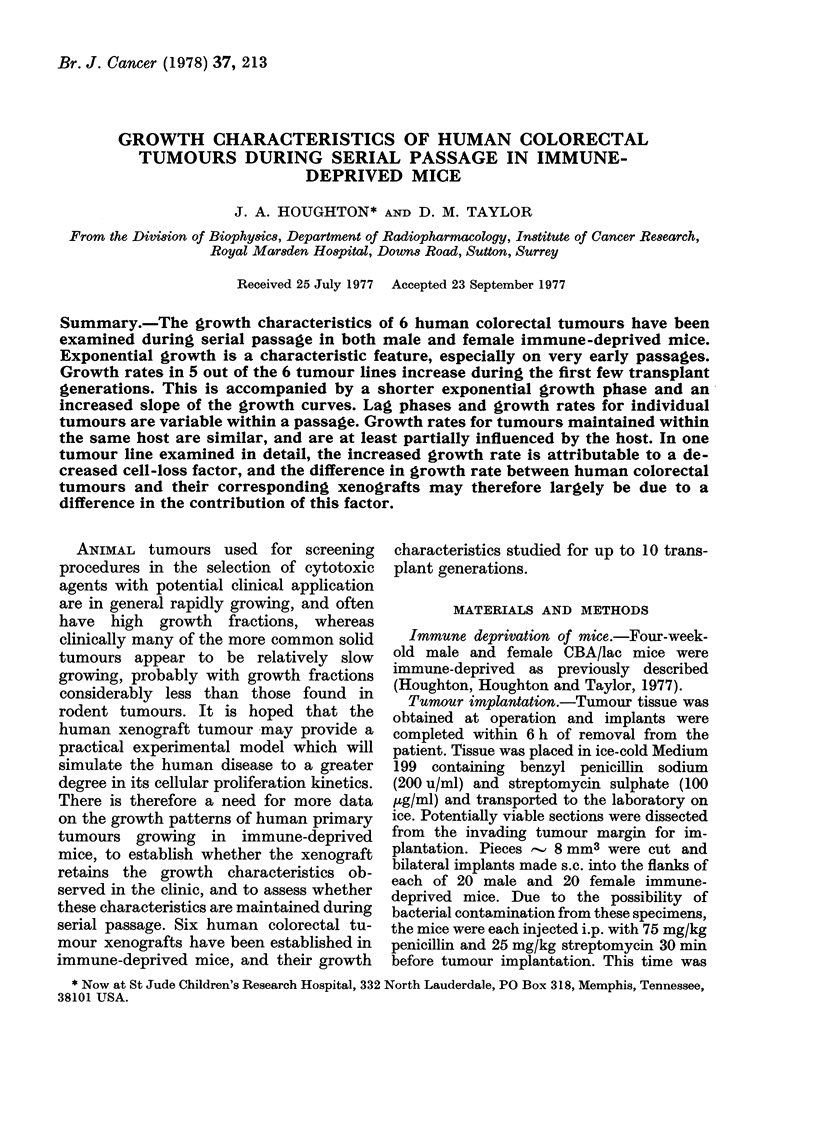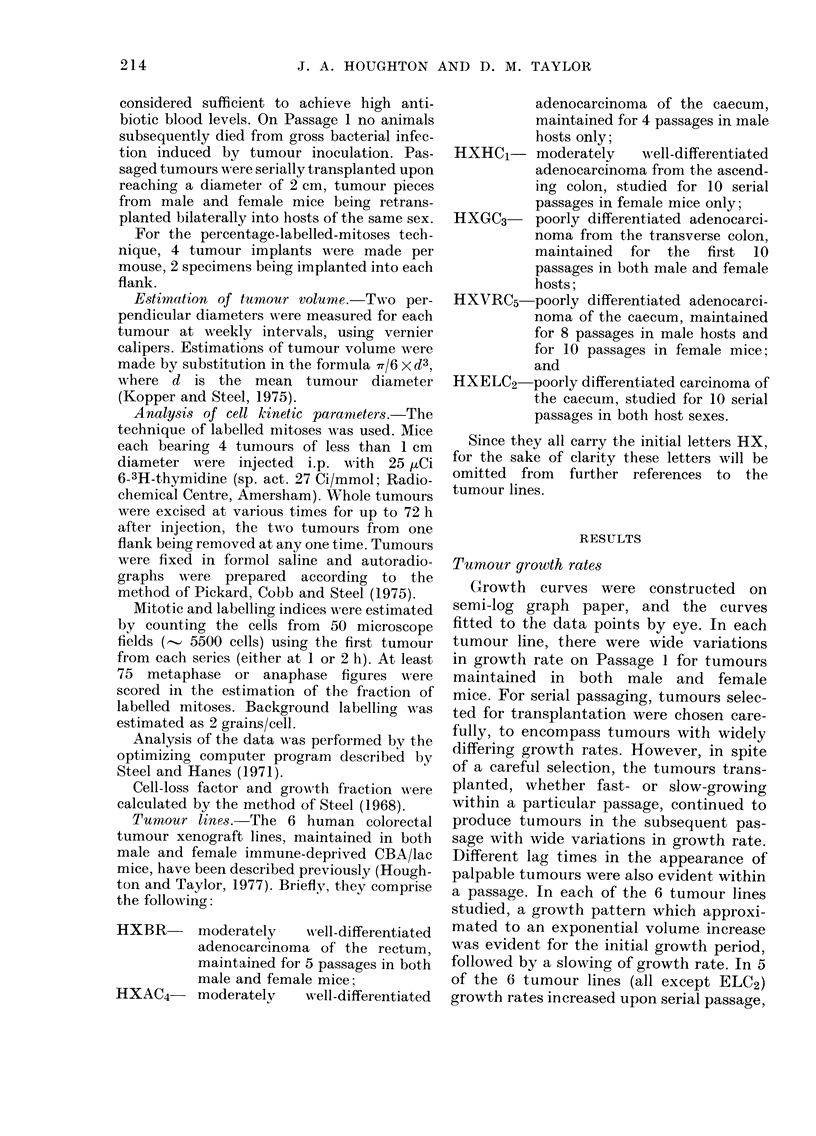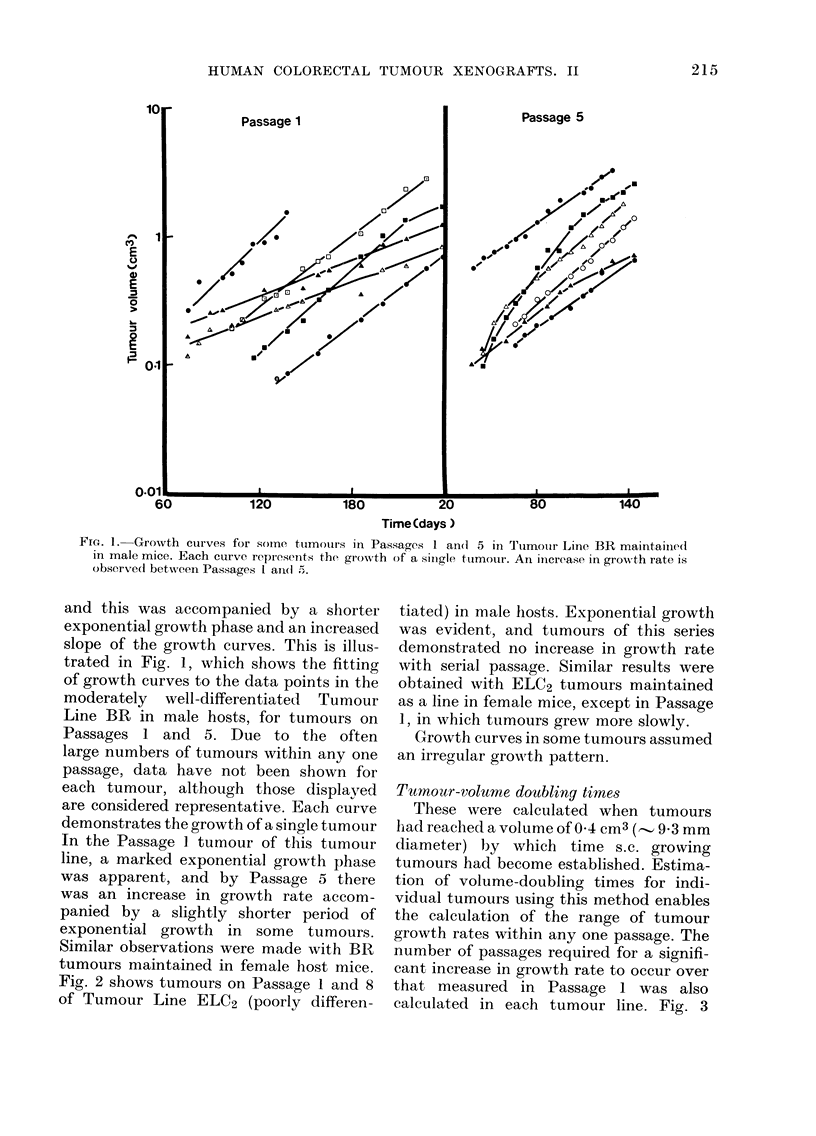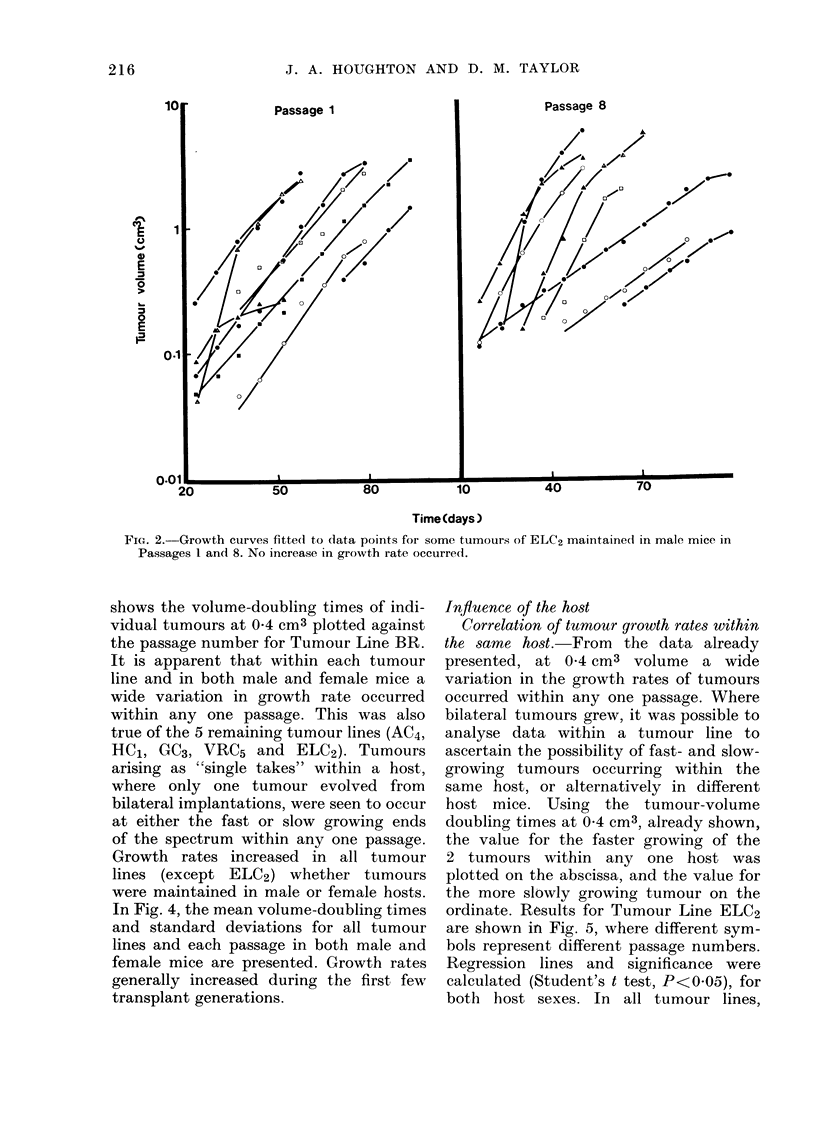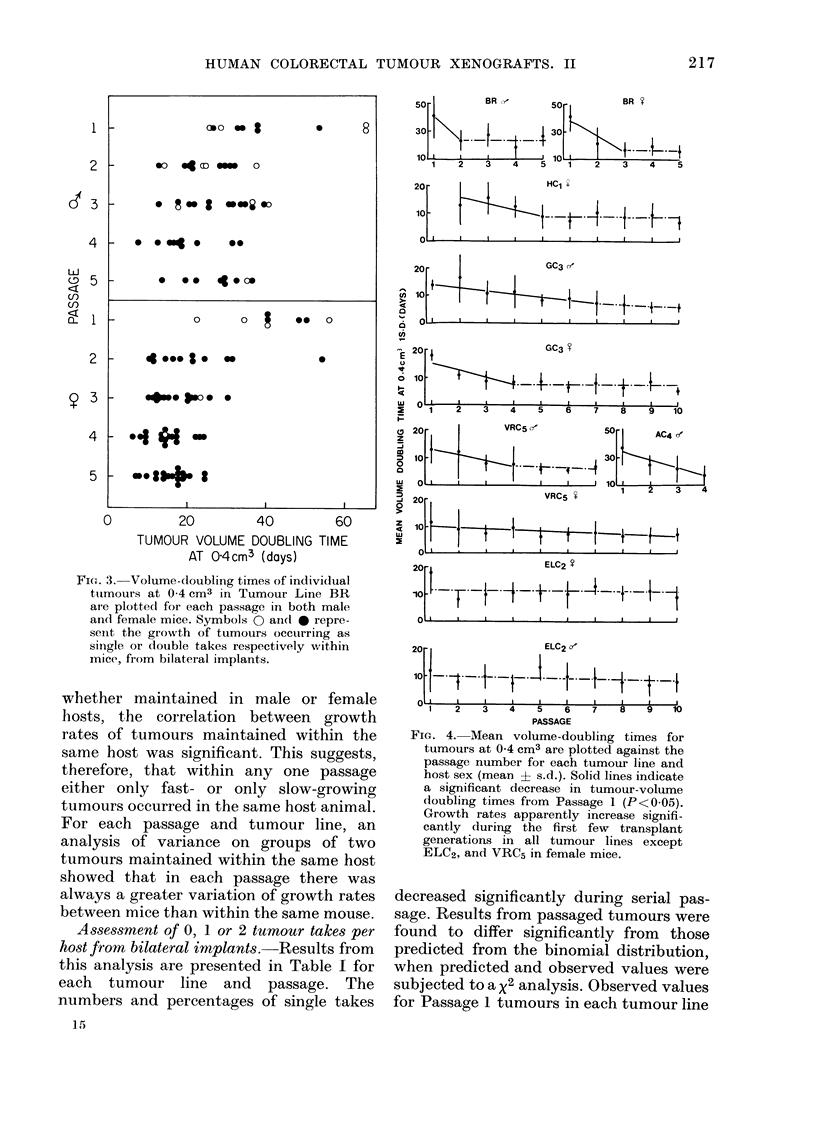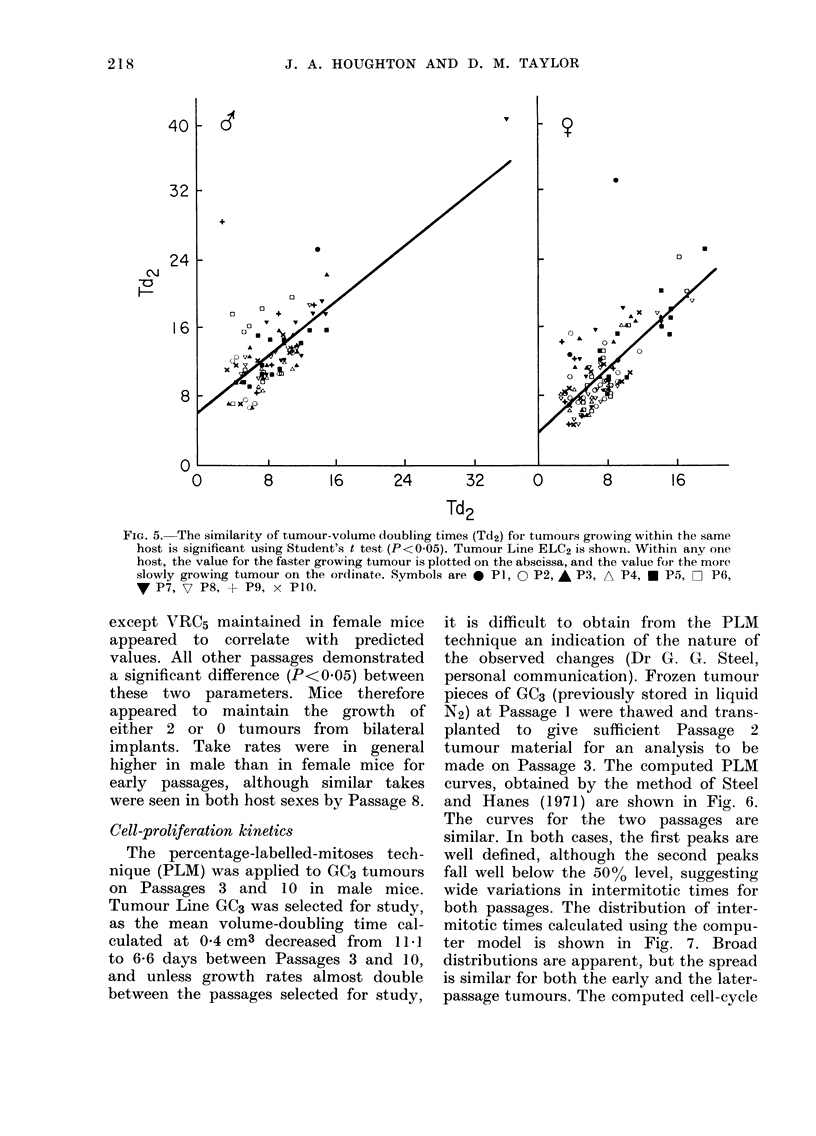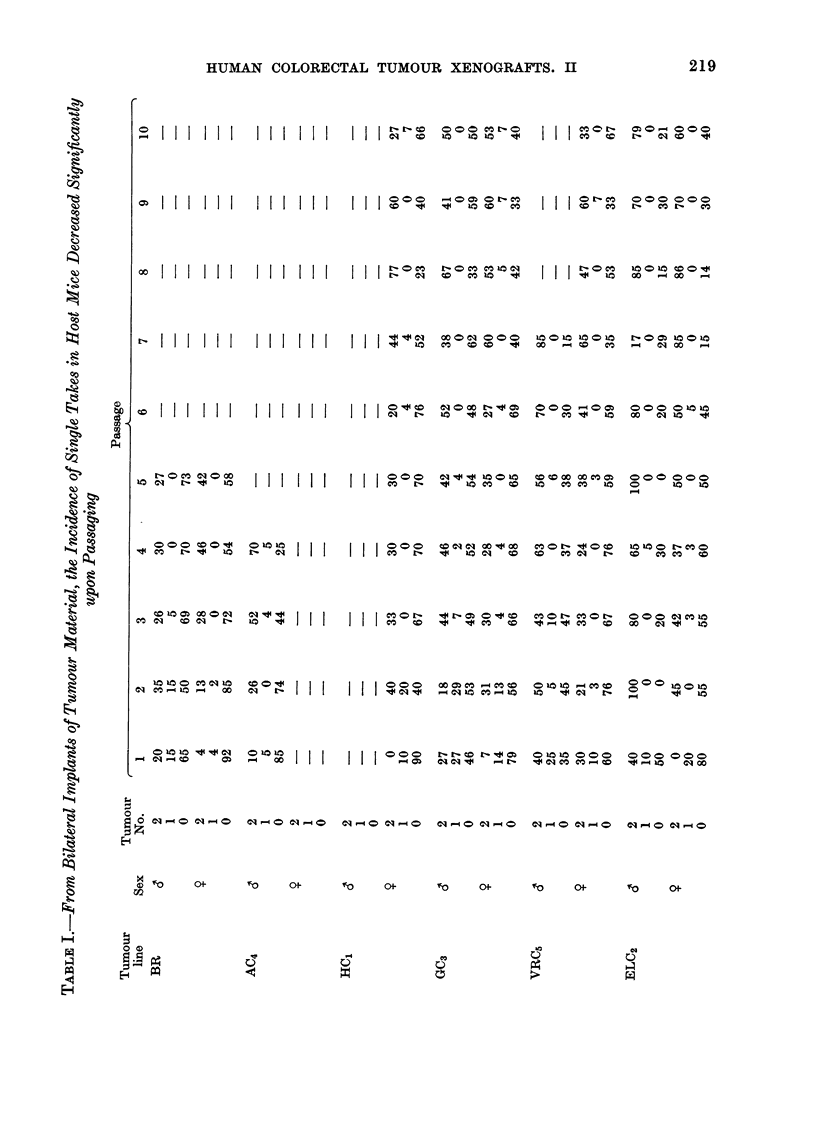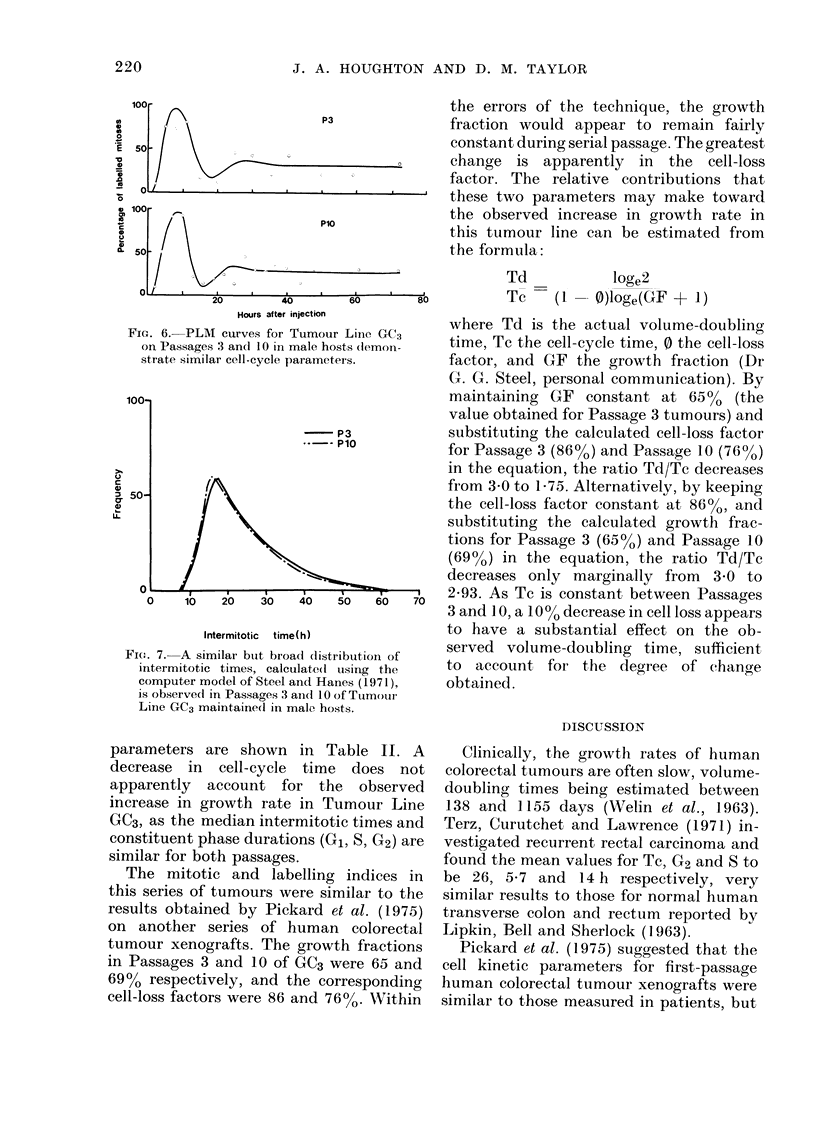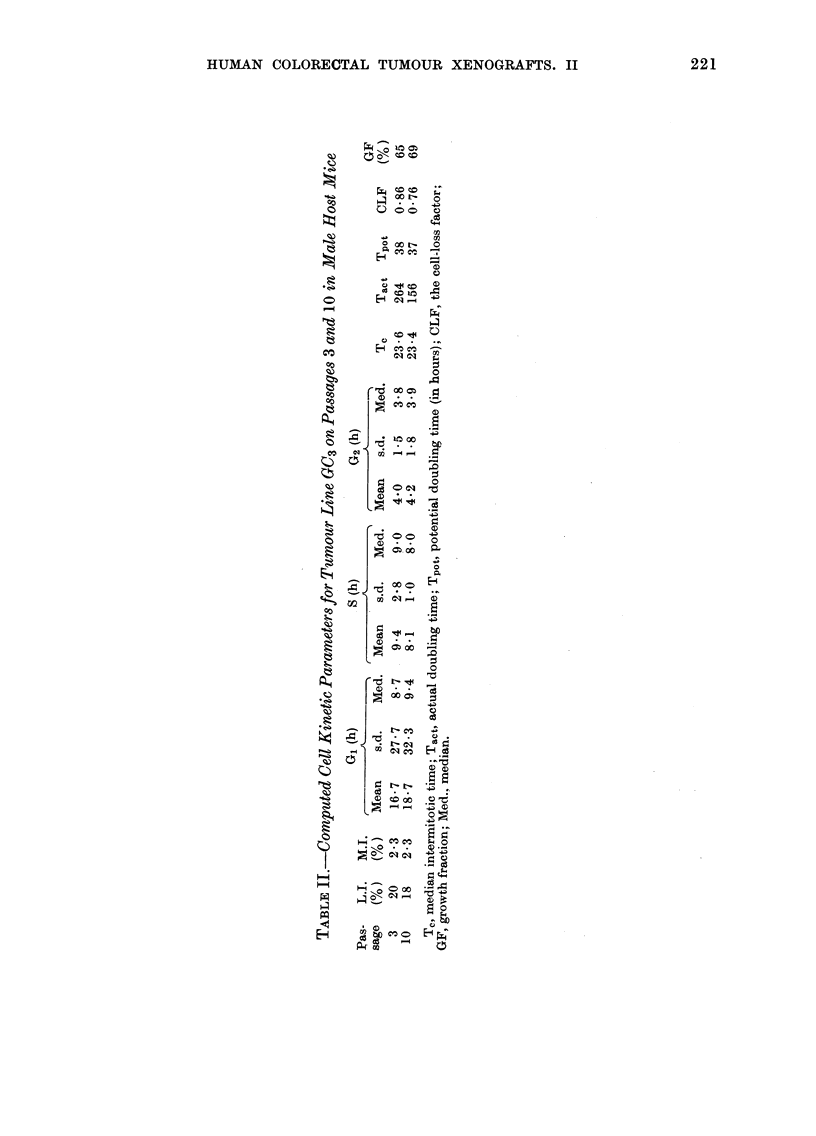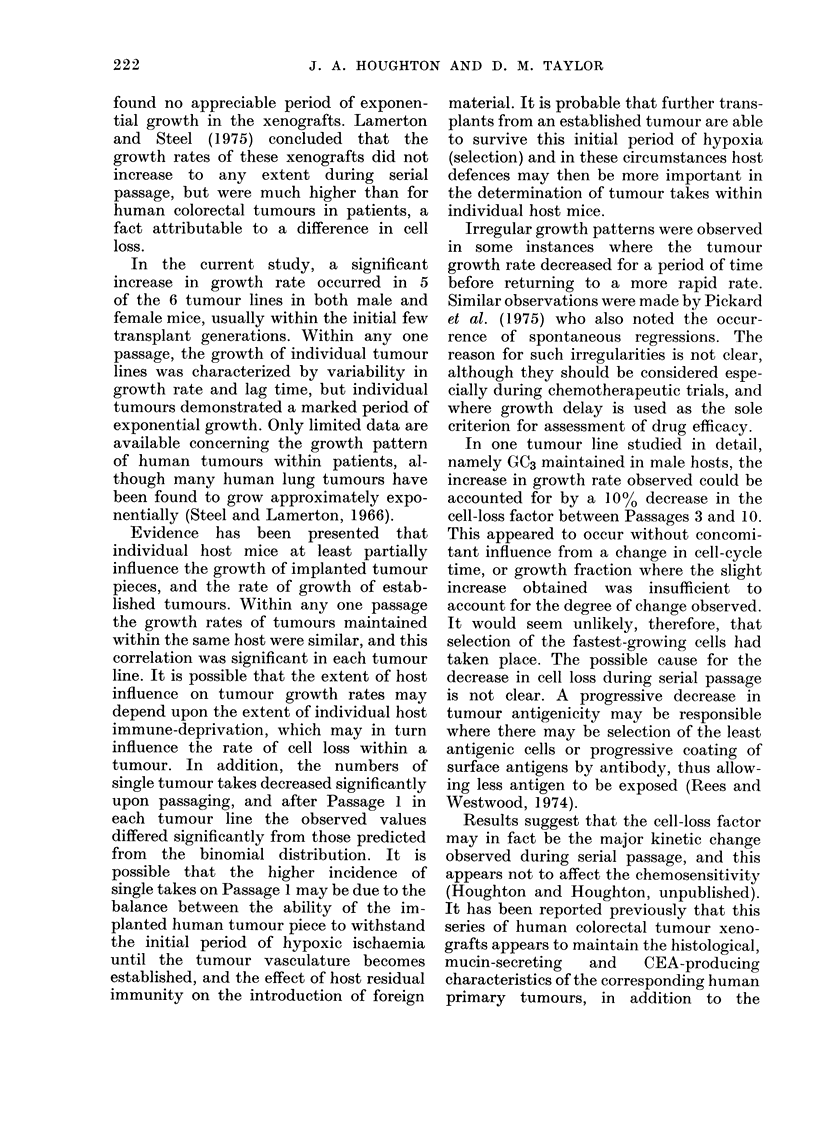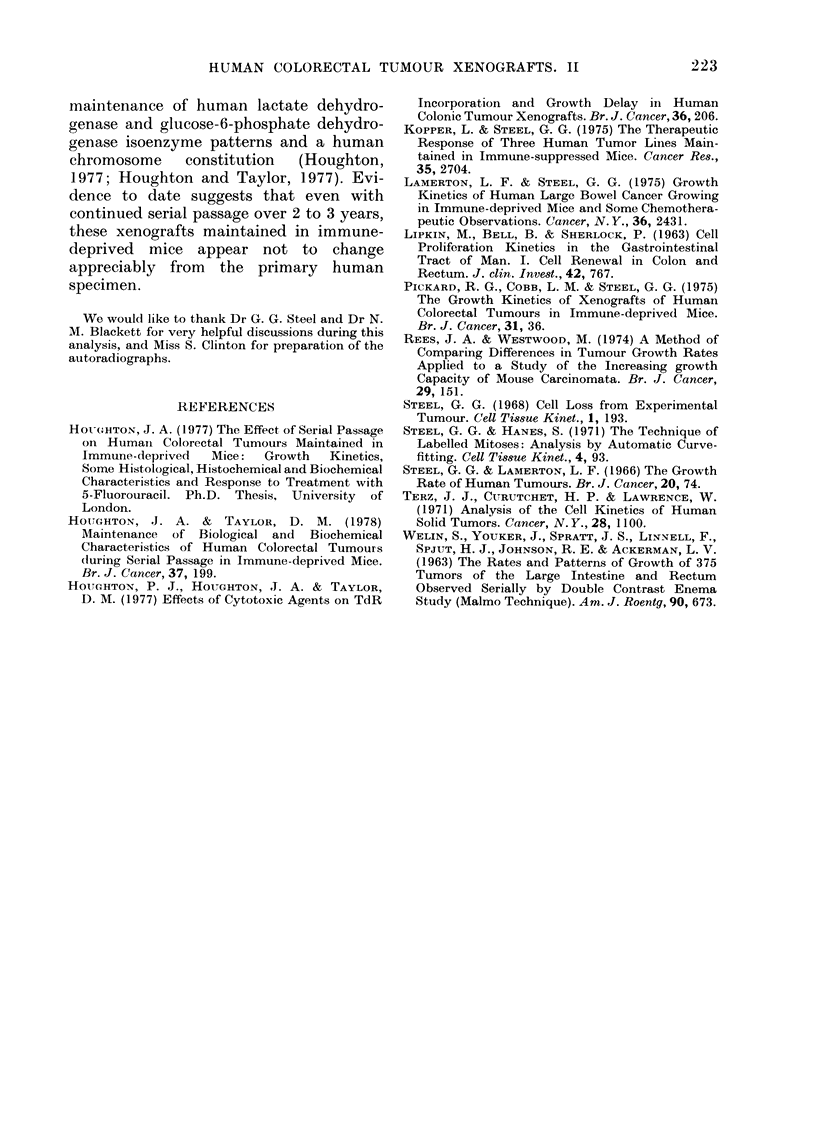# Growth characteristics of human colorectal tumours during serial passage in immune-deprived mice.

**DOI:** 10.1038/bjc.1978.29

**Published:** 1978-02

**Authors:** J. A. Houghton, D. M. Taylor

## Abstract

The growth characteristics of 6 human colorectal tumours have been examined during serial passage in both male and female immune-deprived mice. Exponential growth is a characteristic feature, especially on very early passages. Growth rates in 5 out of the 6 tumour lines increase during the first few transplant generations. This is accompanied by a shorter exponential growth phase and an increased slope of the growth curves. Lag phases and growth rates for individual tumours are variable within a passage. Growth rates for tumours maintained within the same host are similar, and are at least partially influenced by the host. In one tumour line examined in detail, the increased growth rate is attributable to a decreased cell-loss factor, and the difference in growth rate between human colorectal tumours and their corresponding xenografts may therefore largely be due to a difference in the contribution of this factor.


*Br. J. Cancer* (1978) **37**, 213

### GROWTH CHARACTERISTICS OF HUMAN COLORECTAL TUMOURS DURING SERIAL PASSAGE IN IMMUNE-DEPRIVED MICE

J. A. HOUGHTON* AND D. M. TAYLOR

*From the Division of Biophysics, Department of Radiopharmacology, Institute of Cancer Research, Royal Marsden Hospital, Downs Road, Sutton, Surrey*

Received 25 July 1977 Accepted 23 September 1977

**Summary.—The growth characteristics of 6 human colorectal tumours have been examined during serial passage in both male and female immune-deprived mice. Exponential growth is a characteristic feature, especially on very early passages. Growth rates in 5 out of the 6 tumour lines increase during the first few transplant generations. This is accompanied by a shorter exponential growth phase and an increased slope of the growth curves. Lag phases and growth rates for individual tumours are variable within a passage. Growth rates for tumours maintained within the same host are similar, and are at least partially influenced by the host. In one tumour line examined in detail, the increased growth rate is attributable to a decreased cell-loss factor, and the difference in growth rate between human colorectal tumours and their corresponding xenografts may therefore largely be due to a difference in the contribution of this factor.**

ANIMAL tumours used for screening procedures in the selection of cytotoxic agents with potential clinical application are in general rapidly growing, and often have high growth fractions, whereas clinically many of the more common solid tumours appear to be relatively slow growing, probably with growth fractions considerably less than those found in rodent tumours. It is hoped that the human xenograft tumour may provide a practical experimental model which will simulate the human disease to a greater degree in its cellular proliferation kinetics. There is therefore a need for more data on the growth patterns of human primary tumours growing in immune-deprived mice, to establish whether the xenograft retains the growth characteristics observed in the clinic, and to assess whether these characteristics are maintained during serial passage. Six human colorectal tumour xenografts have been established in immune-deprived mice, and their growth characteristics studied for up to 10 transplant generations.

### MATERIALS AND METHODS

*Immune deprivation of mice.*—Four-week-old male and female CBA/lac mice were immune-deprived as previously described (Houghton, Houghton and Taylor, 1977).

*Tumour implantation.*—Tumour tissue was obtained at operation and implants were completed within 6 h of removal from the patient. Tissue was placed in ice-cold Medium 199 containing benzyl penicillin sodium (200 u/ml) and streptomycin sulphate (100 $\mu$g/ml) and transported to the laboratory on ice. Potentially viable sections were dissected from the invading tumour margin for implantation. Pieces $\sim$ 8 mm$^3$ were cut and bilateral implants made s.c. into the flanks of each of 20 male and 20 female immune-deprived mice. Due to the possibility of bacterial contamination from these specimens, the mice were each injected i.p. with 75 mg/kg penicillin and 25 mg/kg streptomycin 30 min before tumour implantation. This time was

* Now at St Jude Children's Research Hospital, 332 North Lauderdale, PO Box 318, Memphis, Tennessee, 38101 USA.

considered sufficient to achieve high antibiotic blood levels. On Passage 1 no animals subsequently died from gross bacterial infection induced by tumour inoculation. Passaged tumours were serially transplanted upon reaching a diameter of 2 cm, tumour pieces from male and female mice being retransplanted bilaterally into hosts of the same sex.

For the percentage-labelled-mitoses technique, 4 tumour implants were made per mouse, 2 specimens being implanted into each flank.

*Estimation of tumour volume.*—Two perpendicular diameters were measured for each tumour at weekly intervals, using vernier calipers. Estimations of tumour volume were made by substitution in the formula $\pi/6 \times d^3$, where $d$ is the mean tumour diameter (Kopper and Steel, 1975).

*Analysis of cell kinetic parameters.*—The technique of labelled mitoses was used. Mice each bearing 4 tumours of less than 1 cm diameter were injected i.p. with 25 $\mu$Ci 6-$^3$H-thymidine (sp. act. 27 Ci/mmol; Radiochemical Centre, Amersham). Whole tumours were excised at various times for up to 72 h after injection, the two tumours from one flank being removed at any one time. Tumours were fixed in formol saline and autoradiographs were prepared according to the method of Pickard, Cobb and Steel (1975).

Mitotic and labelling indices were estimated by counting the cells from 50 microscope fields ($\sim$ 5500 cells) using the first tumour from each series (either at 1 or 2 h). At least 75 metaphase or anaphase figures were scored in the estimation of the fraction of labelled mitoses. Background labelling was estimated as 2 grains/cell.

Analysis of the data was performed by the optimizing computer program described by Steel and Hanes (1971).

Cell-loss factor and growth fraction were calculated by the method of Steel (1968).

*Tumour lines.*—The 6 human colorectal tumour xenograft lines, maintained in both male and female immune-deprived CBA/lac mice, have been described previously (Houghton and Taylor, 1977). Briefly, they comprise the following:

HXBR— moderately well-differentiated adenocarcinoma of the rectum, maintained for 5 passages in both male and female mice;

$HXAC_4$— moderately well-differentiated adenocarcinoma of the caecum, maintained for 4 passages in male hosts only;

$HXHC_1$— moderately well-differentiated adenocarcinoma from the ascending colon, studied for 10 serial passages in female mice only;

$HXGC_3$— poorly differentiated adenocarcinoma from the transverse colon, maintained for the first 10 passages in both male and female hosts;

$HXVRC_5$—poorly differentiated adenocarcinoma of the caecum, maintained for 8 passages in male hosts and for 10 passages in female mice; and

$HXELC_2$—poorly differentiated carcinoma of the caecum, studied for 10 serial passages in both host sexes.

Since they all carry the initial letters HX, for the sake of clarity these letters will be omitted from further references to the tumour lines.

### RESULTS

### *Tumour growth rates*

Growth curves were constructed on semi-log graph paper, and the curves fitted to the data points by eye. In each tumour line, there were wide variations in growth rate on Passage 1 for tumours maintained in both male and female mice. For serial passaging, tumours selected for transplantation were chosen carefully, to encompass tumours with widely differing growth rates. However, in spite of a careful selection, the tumours transplanted, whether fast- or slow-growing within a particular passage, continued to produce tumours in the subsequent passage with wide variations in growth rate. Different lag times in the appearance of palpable tumours were also evident within a passage. In each of the 6 tumour lines studied, a growth pattern which approximated to an exponential volume increase was evident for the initial growth period, followed by a slowing of growth rate. In 5 of the 6 tumour lines (all except $ELC_2$) growth rates increased upon serial passage,

FIG. 1.—Growth curves for some tumours in Passages 1 and 5 in Tumour Line BR maintained in male mice. Each curve represents the growth of a single tumour. An increase in growth rate is observed between Passages 1 and 5.

and this was accompanied by a shorter exponential growth phase and an increased slope of the growth curves. This is illustrated in Fig. 1, which shows the fitting of growth curves to the data points in the moderately well-differentiated Tumour Line BR in male hosts, for tumours on Passages 1 and 5. Due to the often large numbers of tumours within any one passage, data have not been shown for each tumour, although those displayed are considered representative. Each curve demonstrates the growth of a single tumour In the Passage 1 tumour of this tumour line, a marked exponential growth phase was apparent, and by Passage 5 there was an increase in growth rate accompanied by a slightly shorter period of exponential growth in some tumours. Similar observations were made with BR tumours maintained in female host mice. Fig. 2 shows tumours on Passage 1 and 8 of Tumour Line $ELC_2$ (poorly differentiated) in male hosts. Exponential growth was evident, and tumours of this series demonstrated no increase in growth rate with serial passage. Similar results were obtained with $ELC_2$ tumours maintained as a line in female mice, except in Passage 1, in which tumours grew more slowly.

Growth curves in some tumours assumed an irregular growth pattern.

*Tumour-volume doubling times*

These were calculated when tumours had reached a volume of 0·4 $cm^3$ (~ 9·3 mm diameter) by which time s.c. growing tumours had become established. Estimation of volume-doubling times for individual tumours using this method enables the calculation of the range of tumour growth rates within any one passage. The number of passages required for a significant increase in growth rate to occur over that measured in Passage 1 was also calculated in each tumour line. Fig. 3

FIG. 2.—Growth curves fitted to data points for some tumours of $ELC_2$ maintained in male mice in Passages 1 and 8. No increase in growth rate occurred.

shows the volume-doubling times of individual tumours at 0·4 $cm^3$ plotted against the passage number for Tumour Line BR. It is apparent that within each tumour line and in both male and female mice a wide variation in growth rate occurred within any one passage. This was also true of the 5 remaining tumour lines ($AC_4$, $HC_1$, $GC_3$, $VRC_5$ and $ELC_2$). Tumours arising as "single takes" within a host, where only one tumour evolved from bilateral implantations, were seen to occur at either the fast or slow growing ends of the spectrum within any one passage. Growth rates increased in all tumour lines (except $ELC_2$) whether tumours were maintained in male or female hosts. In Fig. 4, the mean volume-doubling times and standard deviations for all tumour lines and each passage in both male and female mice are presented. Growth rates generally increased during the first few transplant generations.

*Influence of the host*

*Correlation of tumour growth rates within the same host.*—From the data already presented, at 0·4 $cm^3$ volume a wide variation in the growth rates of tumours occurred within any one passage. Where bilateral tumours grew, it was possible to analyse data within a tumour line to ascertain the possibility of fast- and slow-growing tumours occurring within the same host, or alternatively in different host mice. Using the tumour-volume doubling times at 0·4 $cm^3$, already shown, the value for the faster growing of the 2 tumours within any one host was plotted on the abscissa, and the value for the more slowly growing tumour on the ordinate. Results for Tumour Line $ELC_2$ are shown in Fig. 5, where different symbols represent different passage numbers. Regression lines and significance were calculated (Student's $t$ test, $P<0.05$), for both host sexes. In all tumour lines,

FIG. 3.—Volume-doubling times of individual tumours at 0·4 cm³ in Tumour Line BR are plotted for each passage in both male and female mice. Symbols ○ and ● represent the growth of tumours occurring as single or double takes respectively within mice, from bilateral implants.

FIG. 4.—Mean volume-doubling times for tumours at 0·4 cm³ are plotted against the passage number for each tumour line and host sex (mean ± s.d.). Solid lines indicate a significant decrease in tumour-volume doubling times from Passage 1 ($P < 0{\cdot}05$). Growth rates apparently increase significantly during the first few transplant generations in all tumour lines except $ELC_2$, and $VRC_5$ in female mice.

whether maintained in male or female hosts, the correlation between growth rates of tumours maintained within the same host was significant. This suggests, therefore, that within any one passage either only fast- or only slow-growing tumours occurred in the same host animal. For each passage and tumour line, an analysis of variance on groups of two tumours maintained within the same host showed that in each passage there was always a greater variation of growth rates between mice than within the same mouse.

*Assessment of* 0, 1 *or* 2 *tumour takes per host from bilateral implants.*—Results from this analysis are presented in Table I for each tumour line and passage. The numbers and percentages of single takes decreased significantly during serial passage. Results from passaged tumours were found to differ significantly from those predicted from the binomial distribution, when predicted and observed values were subjected to a $\chi^2$ analysis. Observed values for Passage 1 tumours in each tumour line

FIG. 5.—The similarity of tumour-volume doubling times ($Td_2$) for tumours growing within the same host is significant using Student's *t* test ($P<0{\cdot}05$). Tumour Line $ELC_2$ is shown. Within any one host, the value for the faster growing tumour is plotted on the abscissa, and the value for the more slowly growing tumour on the ordinate. Symbols are ● P1, ○ P2, ▲ P3, △ P4, ■ P5, □ P6, ▼ P7, ▽ P8, + P9, × P10.

except $VRC_5$ maintained in female mice appeared to correlate with predicted values. All other passages demonstrated a significant difference ($P<0{\cdot}05$) between these two parameters. Mice therefore appeared to maintain the growth of either 2 or 0 tumours from bilateral implants. Take rates were in general higher in male than in female mice for early passages, although similar takes were seen in both host sexes by Passage 8.

*Cell-proliferation kinetics*

The percentage-labelled-mitoses technique (PLM) was applied to $GC_3$ tumours on Passages 3 and 10 in male mice. Tumour Line $GC_3$ was selected for study, as the mean volume-doubling time calculated at 0·4 $cm^3$ decreased from 11·1 to 6·6 days between Passages 3 and 10, and unless growth rates almost double between the passages selected for study, it is difficult to obtain from the PLM technique an indication of the nature of the observed changes (Dr G. G. Steel, personal communication). Frozen tumour pieces of $GC_3$ (previously stored in liquid $N_2$) at Passage 1 were thawed and transplanted to give sufficient Passage 2 tumour material for an analysis to be made on Passage 3. The computed PLM curves, obtained by the method of Steel and Hanes (1971) are shown in Fig. 6. The curves for the two passages are similar. In both cases, the first peaks are well defined, although the second peaks fall well below the 50% level, suggesting wide variations in intermitotic times for both passages. The distribution of intermitotic times calculated using the computer model is shown in Fig. 7. Broad distributions are apparent, but the spread is similar for both the early and the later-passage tumours. The computed cell-cycle

TABLE I.—*From Bilateral Implants of Tumour Material, the Incidence of Single Takes in Host Mice Decreased Significantly upon Passaging*

| Tumour line | Sex | Tumour No. | Passage 1 | 2 | 3 | 4 | 5 | 6 | 7 | 8 | 9 | 10 |
|---|---|---|---|---|---|---|---|---|---|---|---|---|
| BR | ♂ | 2 | 20 | 35 | 26 | 30 | 27 | — | — | — | — | — |
| | | 1 | 15 | 15 | 5 | 0 | 0 | — | — | — | — | — |
| | | 0 | 65 | 50 | 69 | 70 | 73 | — | — | — | — | — |
| | ♀ | 2 | 4 | 13 | 28 | 46 | 42 | — | — | — | — | — |
| | | 1 | 4 | 2 | 0 | 0 | 0 | — | — | — | — | — |
| | | 0 | 92 | 85 | 72 | 54 | 58 | — | — | — | — | — |
| $AC_4$ | ♂ | 2 | 10 | 26 | 52 | 70 | — | — | — | — | — | — |
| | | 1 | 5 | 0 | 4 | 5 | — | — | — | — | — | — |
| | | 0 | 85 | 74 | 44 | 25 | — | — | — | — | — | — |
| | ♀ | 2 | — | — | — | — | — | — | — | — | — | — |
| | | 1 | — | — | — | — | — | — | — | — | — | — |
| | | 0 | — | — | — | — | — | — | — | — | — | — |
| $HC_1$ | ♂ | 2 | — | — | — | — | — | — | — | — | — | — |
| | | 1 | — | — | — | — | — | — | — | — | — | — |
| | | 0 | — | — | — | — | — | — | — | — | — | — |
| | ♀ | 2 | 0 | 40 | 33 | 30 | 30 | 20 | 44 | 77 | 60 | 27 |
| | | 1 | 10 | 20 | 0 | 0 | 0 | 4 | 4 | 0 | 0 | 7 |
| | | 0 | 90 | 40 | 67 | 70 | 70 | 76 | 52 | 23 | 40 | 66 |
| $GC_3$ | ♂ | 2 | 27 | 18 | 44 | 46 | 42 | 52 | 38 | 67 | 41 | 50 |
| | | 1 | 27 | 29 | 7 | 2 | 4 | 0 | 0 | 0 | 0 | 0 |
| | | 0 | 46 | 53 | 49 | 52 | 54 | 48 | 62 | 33 | 59 | 50 |
| | ♀ | 2 | 7 | 31 | 30 | 28 | 35 | 27 | 60 | 53 | 60 | 53 |
| | | 1 | 14 | 13 | 4 | 4 | 0 | 4 | 0 | 5 | 7 | 7 |
| | | 0 | 79 | 56 | 66 | 68 | 65 | 69 | 40 | 42 | 33 | 40 |
| $VRC_5$ | ♂ | 2 | 40 | 50 | 43 | 63 | 56 | 70 | 85 | — | — | — |
| | | 1 | 25 | 5 | 10 | 0 | 6 | 0 | 0 | — | — | — |
| | | 0 | 35 | 45 | 47 | 37 | 38 | 30 | 15 | — | — | — |
| | ♀ | 2 | 30 | 21 | 33 | 24 | 38 | 41 | 65 | 47 | 60 | 33 |
| | | 1 | 10 | 3 | 0 | 0 | 3 | 0 | 0 | 0 | 7 | 0 |
| | | 0 | 60 | 76 | 67 | 76 | 59 | 59 | 35 | 53 | 33 | 67 |
| $ELC_2$ | ♂ | 2 | 40 | 100 | 80 | 65 | 100 | 80 | 17 | 85 | 70 | 79 |
| | | 1 | 10 | 0 | 0 | 5 | 0 | 0 | 0 | 0 | 0 | 0 |
| | | 0 | 50 | 0 | 20 | 30 | 0 | 20 | 29 | 15 | 30 | 21 |
| | ♀ | 2 | 0 | 45 | 42 | 37 | 50 | 50 | 85 | 86 | 70 | 60 |
| | | 1 | 20 | 0 | 3 | 3 | 0 | 5 | 0 | 0 | 0 | 0 |
| | | 0 | 80 | 55 | 55 | 60 | 50 | 45 | 15 | 14 | 30 | 40 |

FIG. 6.—PLM curves for Tumour Line $GC_3$ on Passages 3 and 10 in male hosts demonstrate similar cell-cycle parameters.

FIG. 7.—A similar but broad distribution of intermitotic times, calculated using the computer model of Steel and Hanes (1971), is observed in Passages 3 and 10 of Tumour Line $GC_3$ maintained in male hosts.

parameters are shown in Table II. A decrease in cell-cycle time does not apparently account for the observed increase in growth rate in Tumour Line $GC_3$, as the median intermitotic times and constituent phase durations ($G_1$, S, $G_2$) are similar for both passages.

The mitotic and labelling indices in this series of tumours were similar to the results obtained by Pickard *et al.* (1975) on another series of human colorectal tumour xenografts. The growth fractions in Passages 3 and 10 of $GC_3$ were 65 and 69% respectively, and the corresponding cell-loss factors were 86 and 76%. Within the errors of the technique, the growth fraction would appear to remain fairly constant during serial passage. The greatest change is apparently in the cell-loss factor. The relative contributions that these two parameters may make toward the observed increase in growth rate in this tumour line can be estimated from the formula:

$$\frac{Td}{Tc} = \frac{\log_e 2}{(1 - \emptyset)\log_e(GF + 1)}$$

where Td is the actual volume-doubling time, Tc the cell-cycle time, $\emptyset$ the cell-loss factor, and GF the growth fraction (Dr G. G. Steel, personal communication). By maintaining GF constant at 65% (the value obtained for Passage 3 tumours) and substituting the calculated cell-loss factor for Passage 3 (86%) and Passage 10 (76%) in the equation, the ratio Td/Tc decreases from 3·0 to 1·75. Alternatively, by keeping the cell-loss factor constant at 86%, and substituting the calculated growth fractions for Passage 3 (65%) and Passage 10 (69%) in the equation, the ratio Td/Tc decreases only marginally from 3·0 to 2·93. As Tc is constant between Passages 3 and 10, a 10% decrease in cell loss appears to have a substantial effect on the observed volume-doubling time, sufficient to account for the degree of change obtained.

### DISCUSSION

Clinically, the growth rates of human colorectal tumours are often slow, volume-doubling times being estimated between 138 and 1155 days (Welin *et al.*, 1963). Terz, Curutchet and Lawrence (1971) investigated recurrent rectal carcinoma and found the mean values for Tc, $G_2$ and S to be 26, 5·7 and 14 h respectively, very similar results to those for normal human transverse colon and rectum reported by Lipkin, Bell and Sherlock (1963).

Pickard *et al.* (1975) suggested that the cell kinetic parameters for first-passage human colorectal tumour xenografts were similar to those measured in patients, but

TABLE II.—*Computed Cell Kinetic Parameters for Tumour Line* $GC_3$ *on Passages* 3 *and* 10 *in Male Host Mice*

| Passage | L.I. (%) | M.I. (%) | $G_1$ (h) | | | S (h) | | | $G_2$ (h) | | | $T_c$ | $T_{act}$ | $T_{pot}$ | CLF | GF (%) |
|---|---|---|---|---|---|---|---|---|---|---|---|---|---|---|---|---|
| | | | Mean | s.d. | Med. | Mean | s.d. | Med. | Mean | s.d. | Med. | | | | | |
| 3 | 20 | 2·3 | 16·7 | 27·7 | 8·7 | 9·4 | 2·8 | 9·0 | 4·0 | 1·5 | 3·8 | 23·6 | 264 | 38 | 0·86 | 65 |
| 10 | 18 | 2·3 | 18·7 | 32·3 | 9·4 | 8·1 | 1·0 | 8·0 | 4·2 | 1·8 | 3·9 | 23·4 | 156 | 37 | 0·76 | 69 |

$T_c$, median intermitotic time; $T_{act}$, actual doubling time; $T_{pot}$, potential doubling time (in hours); CLF, the cell-loss factor; GF, growth fraction; Med., median.

found no appreciable period of exponential growth in the xenografts. Lamerton and Steel (1975) concluded that the growth rates of these xenografts did not increase to any extent during serial passage, but were much higher than for human colorectal tumours in patients, a fact attributable to a difference in cell loss.

In the current study, a significant increase in growth rate occurred in 5 of the 6 tumour lines in both male and female mice, usually within the initial few transplant generations. Within any one passage, the growth of individual tumour lines was characterized by variability in growth rate and lag time, but individual tumours demonstrated a marked period of exponential growth. Only limited data are available concerning the growth pattern of human tumours within patients, although many human lung tumours have been found to grow approximately exponentially (Steel and Lamerton, 1966).

Evidence has been presented that individual host mice at least partially influence the growth of implanted tumour pieces, and the rate of growth of established tumours. Within any one passage the growth rates of tumours maintained within the same host were similar, and this correlation was significant in each tumour line. It is possible that the extent of host influence on tumour growth rates may depend upon the extent of individual host immune-deprivation, which may in turn influence the rate of cell loss within a tumour. In addition, the numbers of single tumour takes decreased significantly upon passaging, and after Passage 1 in each tumour line the observed values differed significantly from those predicted from the binomial distribution. It is possible that the higher incidence of single takes on Passage 1 may be due to the balance between the ability of the implanted human tumour piece to withstand the initial period of hypoxic ischaemia until the tumour vasculature becomes established, and the effect of host residual immunity on the introduction of foreign material. It is probable that further transplants from an established tumour are able to survive this initial period of hypoxia (selection) and in these circumstances host defences may then be more important in the determination of tumour takes within individual host mice.

Irregular growth patterns were observed in some instances where the tumour growth rate decreased for a period of time before returning to a more rapid rate. Similar observations were made by Pickard *et al.* (1975) who also noted the occurrence of spontaneous regressions. The reason for such irregularities is not clear, although they should be considered especially during chemotherapeutic trials, and where growth delay is used as the sole criterion for assessment of drug efficacy.

In one tumour line studied in detail, namely $GC_3$ maintained in male hosts, the increase in growth rate observed could be accounted for by a 10% decrease in the cell-loss factor between Passages 3 and 10. This appeared to occur without concomitant influence from a change in cell-cycle time, or growth fraction where the slight increase obtained was insufficient to account for the degree of change observed. It would seem unlikely, therefore, that selection of the fastest-growing cells had taken place. The possible cause for the decrease in cell loss during serial passage is not clear. A progressive decrease in tumour antigenicity may be responsible where there may be selection of the least antigenic cells or progressive coating of surface antigens by antibody, thus allowing less antigen to be exposed (Rees and Westwood, 1974).

Results suggest that the cell-loss factor may in fact be the major kinetic change observed during serial passage, and this appears not to affect the chemosensitivity (Houghton and Houghton, unpublished). It has been reported previously that this series of human colorectal tumour xenografts appears to maintain the histological, mucin-secreting and CEA-producing characteristics of the corresponding human primary tumours, in addition to the maintenance of human lactate dehydrogenase and glucose-6-phosphate dehydrogenase isoenzyme patterns and a human chromosome constitution (Houghton, 1977; Houghton and Taylor, 1977). Evidence to date suggests that even with continued serial passage over 2 to 3 years, these xenografts maintained in immune-deprived mice appear not to change appreciably from the primary human specimen.

We would like to thank Dr G. G. Steel and Dr N. M. Blackett for very helpful discussions during this analysis, and Miss S. Clinton for preparation of the autoradiographs.

### REFERENCES

HOUGHTON, J. A. (1977) The Effect of Serial Passage on Human Colorectal Tumours Maintained in Immune-deprived Mice: Growth Kinetics, Some Histological, Histochemical and Biochemical Characteristics and Response to Treatment with 5-Fluorouracil. Ph.D. Thesis, University of London.

HOUGHTON, J. A. & TAYLOR, D. M. (1978) Maintenance of Biological and Biochemical Characteristics of Human Colorectal Tumours during Serial Passage in Immune-deprived Mice. *Br. J. Cancer*, **37**, 199.

HOUGHTON, P. J., HOUGHTON, J. A. & TAYLOR, D. M. (1977) Effects of Cytotoxic Agents on TdR Incorporation and Growth Delay in Human Colonic Tumour Xenografts. *Br. J. Cancer*, **36**, 206.

KOPPER, L. & STEEL, G. G. (1975) The Therapeutic Response of Three Human Tumor Lines Maintained in Immune-suppressed Mice. *Cancer Res.*, **35**, 2704.

LAMERTON, L. F. & STEEL, G. G. (1975) Growth Kinetics of Human Large Bowel Cancer Growing in Immune-deprived Mice and Some Chemotherapeutic Observations. *Cancer, N.Y.*, **36**, 2431.

LIPKIN, M., BELL, B. & SHERLOCK, P. (1963) Cell Proliferation Kinetics in the Gastrointestinal Tract of Man. I. Cell Renewal in Colon and Rectum. *J. clin. Invest.*, **42**, 767.

PICKARD, R. G., COBB, L. M. & STEEL, G. G. (1975) The Growth Kinetics of Xenografts of Human Colorectal Tumours in Immune-deprived Mice. *Br. J. Cancer*, **31**, 36.

REES, J. A. & WESTWOOD, M. (1974) A Method of Comparing Differences in Tumour Growth Rates Applied to a Study of the Increasing growth Capacity of Mouse Carcinomata. *Br. J. Cancer*, **29**, 151.

STEEL, G. G. (1968) Cell Loss from Experimental Tumour. *Cell Tissue Kinet.*, **1**, 193.

STEEL, G. G. & HANES, S. (1971) The Technique of Labelled Mitoses: Analysis by Automatic Curve-fitting. *Cell Tissue Kinet.*, **4**, 93.

STEEL, G. G. & LAMERTON, L. F. (1966) The Growth Rate of Human Tumours. *Br. J. Cancer*, **20**, 74.

TERZ, J. J., CURUTCHET, H. P. & LAWRENCE, W. (1971) Analysis of the Cell Kinetics of Human Solid Tumors. *Cancer, N.Y.*, **28**, 1100.

WELIN, S., YOUKER, J., SPRATT, J. S., LINNELL, F., SPJUT, H. J., JOHNSON, R. E. & ACKERMAN, L. V. (1963) The Rates and Patterns of Growth of 375 Tumors of the Large Intestine and Rectum Observed Serially by Double Contrast Enema Study (Malmo Technique). *Am. J. Roentg*, **90**, 673.